# Identification and epidemiological analysis of a putative novel hantavirus in Australian flying foxes

**DOI:** 10.1007/s11262-024-02113-3

**Published:** 2024-10-11

**Authors:** Craig S. Smith, Darren J. Underwood, Anita Gordon, Michael J. Pyne, Anna Smyth, Benjamin Genge, Luke Driver, David G. Mayer, Jane Oakey

**Affiliations:** 1 Biosecurity Sciences Laboratory, Department of Agriculture and Fisheries, Biosecurity Queensland, Brisbane, QLD Australia; 2 Currumbin Wildlife Hospital Foundation, Currumbin, QLD Australia

**Keywords:** Bat, Epidemiology, Hantavirus, Infection dynamics, Mobatvirus, Pteropus

## Abstract

In July 2017, an investigation into the cause of neurological signs in a black flying fox (*Pteropus alecto*, family *Pteropodidae*) identified a putative novel hantavirus (Robina virus, ROBV, order *Bunyavirales*, family *Hantaviridae*, genus *Mobatvirus*) in its brain. Analysis of the evolutionary relationship between other hantaviruses using maximum-likelihood, a systematic Bayesian clustering approach, and a minimum spanning tree, all suggest that ROBV is most closely related to another *Mobatvirus*, Quezon virus, previously identified in the lung of a Philippine frugivorous bat (*Rousettus amplexicaudatus*, also family *Pteropodidae*). Subsequently, between March 2018 and October 2023, a total of 495 bats were opportunistically screened for ROBV with an experimental qRT-PCR. The total prevalence of ROBV RNA detected in *Pteropus spp.* was 4.2% (95% CI 2.8–6.4%). Binomial modelling identified that there was substantial evidence supporting an increase (*P* = 0.033) in the detection of ROBV RNA in bats in 2019 and 2020 suggesting of a possible transient epidemic. There was also moderate evidence to support the effect of season (*P* = 0.064), with peak detection in the cooler seasons, autumn, and winter, possibly driven by physiological and ecological factors similar to those already identified for other bat-borne viruses. This is Australia’s first reported putative hantavirus and its identification could expand the southern known range of hantaviruses in Australasia.

**Supplementary Information:**

The online version contains supplementary material available at 10.1007/s11262-024-02113-3.

## Introduction

Rodents, belonging to the order Rodentia (families *Muridae* and *Cricetidae*), have long been recognised as hosts of hantaviruses (order *Bunyavirales*, family *Hantaviridae*), the etiological agents of serious human illnesses including: haemorrhagic fever with renal syndrome (HFRS) in Asia and Europe, and hantavirus cardiopulmonary syndrome (HCPS) in the Americas [1–3]. Historically, a close association between rodents and hantaviruses was observed, leading to the hypothesis that the two had co-evolved over millions of years [4, 5]. However, recent discoveries of other genetically distinct hantaviruses (genera *Loanvirus*, *Mobatvirus* and *Thottimvirus*), identified in moles (order Eulipotyphla, subfamilies *Scalopinae* and *Talpinae*), shrews (order Eulipotyphla, subfamilies *Crocidurinae*, *Myosoticinae*, and *Soricinae*) and bats (order Chiroptera, families *Hipposideridae*, *Molossidae, Nycteridae, Phyllostomidae*, *Pteropodidae*, *Rhinolophidae* and *Vespertilionidae*), have challenged this hypothesis [4, 6–13]. The basal positioning of bat- and mole-borne mobatviruses and selected shrew-borne thottimviruses (from maximum-likelihood and Bayesian analysis), suggests that bats, moles and/or shrews, rather than rodents may have served as the mammalian hosts of ancient hantaviruses [5, 14, 15].

Bats possess unique characteristics that allow them to maintain and transmit viruses [16]. These include flight, their ability to enter into torpor or hibernate, a relatively long-life span, roosting in large, dense groups and a unique immunology [6, 9, 16–18]. Additionally, being ancient (evolving 50–65 million years ago), bats have changed relatively little in comparison to other mammals [16, 19]. This conservation of physiological attributes, including cellular receptors and biochemical pathways, could enhance the transmission of bat-borne viruses to other mammals which share related receptors and pathways [16].

The hantavirus virion is enveloped, small (80–120 nm) and generally spherical or pleomorphic in shape. The negative-sense, single stranded RNA genome is tri-segmented and approximately 12,000 nucleotides in length [20–22]. The segments, small (S), medium (M) and large (L) encode for a nucleocapsid protein (N), envelope glycoproteins that are post-translationally cleaved (Gn and Gc), and the RNA-dependent RNA polymerase (RdRp) [23, 24]. In a revised taxonomic classification, using concatenated S and M amino acid coding regions, hantaviruses are now classified into four new genera [25]. Members of *Orthohantavirus* (which include the only hantaviruses shown to cause serious human illness) are predominantly hosted by rodents, with additional detections in moles and shrews. *Thottimvirus* are hosted by shrews, and both *Loanvirus* and *Mobatvirus* are hosted predominantly by bats [14, 25]. However, studies of genetically diverse bat-borne hantaviruses have been hampered by the limited availability of complete genomes. Partial coding sequences produce discrepancies in phylogenetic analysis and limit development of diagnostic tools [9, 24, 26, 27].

Here, we report the complete coding sequence of the first putative hantavirus identified in Australia, contributing to the advancement of hantavirus taxonomy and expanding the known geographic range of the *Mobatvirus* genus. Additionally, we describe the development of an experimental qRT-PCR, which was subsequently used in passive molecular surveillance of flying foxes to identify potential risk factors for detection.

## Materials and methods

### Sample submission

In July 2017, a black flying fox (*Pteropus alecto*, family *Pteropodidae*), a large frugivorous bat with a history of neurological signs was admitted to a wildlife hospital (Queensland, Australia). The bat was euthanised and submitted to the Biosecurity Sciences Laboratory (BSL, Queensland, Australia) where the rabies-like Australian bat lyssavirus (ABLV) was excluded using a modified qRT-PCR [28]. Histopathology of the brain identified a mild encephalitis. Additional bats, also submitted to BSL for ABLV exclusion, were included in this investigation. Experienced veterinary pathologists identified the species of bats using available keys [19], and performed necropsies to remove the brain. Occasionally, and on an ad hoc basis, additional tissues not required for ABLV exclusion were also collected.

### High throughput sequencing

For further investigations using high throughput sequencing (HTS), total RNA was extracted from the brain using the RNeasy Mini Kit (QIAGEN) and an on-column RNase-free DNAase digestion (QIAGEN), both performed following manufacturer’s instructions, except for a final elution volume of 30 μL. The RNA concentration was measured using a Qubit Fluorometer with the Qubit RNA HS Assay Kit (ThermoFisher Scientific). The sequence library was prepared using the TruSeq Stranded mRNA Library Preparation Kit (Illumina) following the manufacturer’s instructions except for the substitution of Oligo-dT capture beads with those from the Ribo-ZeroTM rRNA Removal Kit (Human/Mouse/Rat, Illumina) to deplete the ribosomal RNA. The size and purity of the sequence library was quantified using the 2200 TapeStation (Agilent), with the final concentration quantified using the Qubit dsDNA HS Assay Kit (ThermoFisher Scientific). The library was sequenced on a NextSeq 500 Sequencing Platform using a NextSeq Mid Output Kit v2 300 (Illumina).

Quality control and FASTQ file generation was initially performed using the online server BaseSpace Sequence Hub (Illumina). Additional trimming was performed using Geneious (Biomatters) [29], and the plugin BBDuk (Bushnell, B., https://sourceforge.net/projects/bbmap/). Continuing with Geneious, 12.6 million paired reads were mapped to a viral genome resource (NCBI) [30], and fine-tuned with additional mapping iterations.

### Phylogeny

The evolutionary relationship between the putative novel hantavirus identified using HTS, and other hantaviruses, previously classified by International Committee on Taxonomy of Viruses (ICTV) [25, 31], was performed using the concatenated amino acid sequence for the complete coding sequence of both the S and M segments, (Table 1, Supplementary material). To maximise the number of available reference sequences the L segment was excluded, aligning analysis with previous studies [25]. A maximum-likelihood tree was created using a method described by Hall [32]. Briefly, codons for the concatenated nucleotide sequences were aligned using Muscle in the program MEGA X [33], and the sequences translated into amino acids. The most appropriate substitution model was identified by testing all available models and selecting the one that had the best fit for the available data, as measured by the Bayesian information criterion [33]. The chosen model (Le Gascuel with gamma distribution and a proportion of invariable sites, LG + G + I) was bootstrapped 1,000 times to estimate the reliability of the nodes. Nodes < 0.7 were considered to be unreliable [32].

**Table 1:** Descriptive and univariate statistics from 495 Australian bats tested for a putative novel hantavirus Robina virus (ROBV) by an experimental qRT-PCR

Variable	Category	Detected (Total)	Prevalence (95%CI)	Adjusted (SE)	*P*
Year	2018	0 (53)	0.0 (0.0–5.8)	0.0 (0.0)	0.033
	2019	10 (180)	5.6 (2.9–10.0)	5.6 (1.8)	
	2020	7 (85)	8.2 (3.8–16.3)	7.7 (2.9)	
	2021	2 (49)	4.1 (0.4–14.5)	13.1 (12.9)	
	2022	1 (86)	1.2 (0.1–6.9)	1.1 (1.1)	
	2023	1 (62)	1.6 (0.1–9.4)	1.1 (1.1)	
Season	Spring	5 (177)	2.8 (1.0–6.6)	3.3 (1.2)	0.064
	Summer	3 (138)	2.2 (0.5–6.5)	1.4 (1.0)	
	Autumn	7 (100)	7.0 (3.2–14.0)	8.4 (3.6)	
	Winter	6 (101)	5.9 (2.5–12.6)	8.5 (3.8)	
Region	Central Queensland	1 (20)	5.0 (0.1–25.4)	6.2 (5.9)	0.281
	Darling Downs	2 (23)	8.7 (1.3–28.0)	12.7 (7.8)	
	Far North Queensland	0 (28)	0.0 (0.0–10.5)	0.0 (0.1)	
	North Queensland	1 (31)	3.2 (0.1–17.6)	4.0 (4.1)	
	South East Queensland	17 (389)	4.4 (2.7–6.9)	5.0 (1.5)	
	Wide BayBurnett	0 (25)	0.0 (0.0–11.6)	0.0 (0.1)	
Species	Insectivorous bat	0 (41)	0.0 (0.0–7.4)	0.0 (0.1)	0.317
	*Pteropus alecto*	16 (332)	4.8 (2.9–7.7)	5.3 (1.6)	
	*Pteropus conspicillatus*	0 (16)	0.0 (0.0–17.1)	0.0 (0.4)	
	*Pteropus poliocephalus*	2 (52)	3.96 (0.3–13.7)	4.4 (3.1)	
	*Pteropus scapulatus*	3 (75)	4.0 (0.9–11.6)	5.1 (3.0)	
Total		21 (495)	4.2 (2.8–6.4)		

Adjusted prevalence and standard error from a fitted binomial logistic regression model (Year + Season)

### Hierarchical clustering

Hierarchical clustering or inference of population structure of hantaviruses was performed using the concatenated amino acid sequences, and a systematic Bayesian clustering approach applying Markov Chain Monte Carlo (MCMC) estimation, available in the program STRUCTURE [34]. The following parameters were adopted; no admixture, independent allele frequencies, a 10,000 burn-in period, 100,000 MCMC steps, assumed population number (*K*) 1 to 10, and 30 iterations [35]. The appropriate *K* value was identified using the rate of change in the log probability of data between successive *K* values [36], implemented in the program STRUCTURE HARVESTER [37]. The posterior probability that an individual hantavirus belonged to population *k* was calculated using the ‘*Greedy* algorithm’ in the program CLUMPP [35, 38].

### Minimum spanning tree

A minimum spanning tree (MST) that connects hantaviruses by the minimum genetic distance, was created using multilocus sequence typing (MLST) of the concatenated amino acid sequences with the eBURST algorithm implemented in the program PhyloViZ [39]. The reliability of the tree was tested in the program MSTgold [40].

### qRT-PCR

An experimental qRT-PCR targeting a 78 bp region of the S segment (MK165655, nucleotide position 852-909) of the putative novel hantavirus identified using HTS. The nucleocapsid gene encoded for by the S segment is a well-established target for molecular assays of orthohantaviruses and primers were designed using Primer3 software Version 4.1.0 [41–44]. Specificity of the probe (5’-[FAM]-AGGGTGTAAGCTTGTTAAAGACA-[TAMRA]-3’, 873-895) and primers (FWD 5’-CTACGAAGCTGCAAAGGTGG-3’, 852-871 and REV 5’-CAAGCAAATACCCAAGGAGCA-3’, 909-929) were assessed in silico using Primer-BLAST (NCBI), and against other viral RNA held at BSL (Hendra virus genotype 1, Hendra virus genotype 2, ABLV, Kunjin virus, Murray Valley encephalitis virus, Ross River virus and Japanese encephalitis virus). The brains of bats (both frugivorous and insectivorous) routinely submitted to BSL for ABLV exclusion were opportunistically screened. Nucleic acid extraction was performed using either the EZ-1 Advanced XL (QIAGEN) or KingFisher DUO Prime (Thermo Fisher Scientific) following manufacturer’s instructions. Template RNA (5 µL) was added to 20 µL master mix containing either SuperScript III Platinum One-Step qRT-PCR Kit (Invitrogen) or Path-ID Multiplex One-Step RT-PCR Kit (Applied Biosystems), with primers at a final concentration of 1.6 µM each, and probe at a final concentration of 0.2 µM as determined by checkerboard optimisation. Thermal cycling was performed with an initial RT step of 50 °C for 15 min, an initial denaturation of 2 min (SuperScript III) or 10 min (Path-ID) prior to 2-step cycling at 95 °C for 15 s and 60 °C for 30 s, repeated 44 times. Cycling was performed on the Rotor-Gene Q real-time PCR cycler (QIAGEN) and results were analysed with the Rotor-Gene Q software (QIAGEN). Hantavirus RNA was considered to have been detected when samples reported a cycle threshold (Ct) of < 40.

### Confirmatory nested RT-PCR

A nested RT-PCR targeting a 498 bp region (MK165655, nucleotide position 687-1185, 381 bp internal, 744-1125) that flanked the expected qRT-PCR amplicon, was used to confirm the nucleotide sequence of hantavirus RNA detected by the qRT-PCR. It was also designed using Primer3 software Version 4.1.0 [41], and the specificity of the primary (FWD 5’-CCTTGCGGAGAAATGGGATG-3’, nucleotide position 687-707, and REV 5’-TGGTCTGTCATTGCTTTGCC-3’, 1165-1185) and secondary primers (FWD 5’-CATAGATGCTGGCCCCACTA-3’, 744-764, and REV 5’-TGTATTCCCATGGACTGCGT-3’, 1105-1125) assessed in-silico using Primer-BLAST (NCBI). Nucleic acid previously extracted for the experimental qRT-PCR was used in the nested RT-PCR. Five (5) µL of nucleic acid template was added to a 20 µL primary reaction master mix containing SuperScript III One-Step RT-PCR Kit (Invitrogen) as per the manufacturer’s instructions scaled to a final volume of 25 uL. Thermal cycling conditions for the primary reaction consisted of a 30 min reverse transcription step at 55 °C, a denaturation step at 95 °C for 2 min followed by 35 cycles of 95 °C for 15 s, 55 °C for 30 s and 72 °C for 45 s. Following this, a final extension of 5 min at 72 °C. Five (5) µL of the primary PCR product was used as the template in the 25 µL nested PCR master mix containing MyTaq HS red mix (Bioline) as per the manufacturer’s instructions. The nested cycling conditions consisted of a 5 min denaturation step at 95 °C prior to 35 cycles of 95 °C for 15 s, 60 °C for 30 s and 72 °C for 45 s. A final extension of 72 °C for 5 min. Products from the nested PCR were purified using AMPure XP Beads-Based Reagent (Beckman Coulter), sequenced using BigDye 3.1 (Applied Biosystems), cleaned with Sephadex (Sigma-Aldrich), and resolved using a 3500 Series Genetic Analyser (Applied Biosystems), all following manufacturer’s instructions. Sequences were proofread and examined using Geneious (Biomatters) and Sequencher (Gene Codes).

### Statistics

Descriptive statistics, including mean prevalence and the calculations of 95% confidence intervals for binomial populations [45], were calculated in Excel. Identification of risk factors was performed using a generalised linear model [46], with the binomial distribution and logit link, using Genstat (VSN International, 2023). Adjusted mean proportions and their standard errors were estimated. P values were also estimated, but rather than focusing solely on their threshold for significance, subjective assessment terms based on the strength of the evidence were used: minimal, moderate, substantial and overwhelming evidence.

### Virus isolation

Virus isolation was attempted using both Vero and BSR cells lines on a sample of fresh brain homogenised in virus transport media, with sterile sand and a mortar and pestle. Cells were maintained for seven days in Eagle’s minimal essential medium (Merck Life Science Pty Ltd) supplemented with 10% heat inactivated foetal bovine serum, 2 mM L-glutamine, 0.2% penicillin–streptomycin sulphate, 0.2% fungizone at 37 °C in the presence of 5% CO_2_. Two additional passages were performed.

### Immunohistochemical staining

Immunohistochemical staining was attempted on brain tissue that had been fixed in 10% formalin for 48 h, dehydrated, paraffin-embedded, and 4 µm sections cut onto slides. Sections were then deparaffinised and pretreated in microwave oven in citrate buffer pH 6.0. Polyclonal antibodies against Puumala virus and Hantaan virus, were used in 1∶100 dilution.

## Results

### High throughput sequencing

Assembly of the HTS data (with a Phred quality score > 30), identified the complete coding sequences for three segments (S, M and L), of a putative novel hantavirus (MK165653–MK165655) [47]. The segments had a read depth of 3147, 4211 and 1464, and had greatest nucleotide sequence similarity (70%, 64% and 72%), and amino acid sequence similarity (80%, 63% and 78%) to the S, M and L segments from the bat-borne *mobatvirus*, Quezon virus (QZNV), identified in the lung of a *Rousettus amplexicaudatus* (a frugivorous bat), from the Philippines in 2016 [13]. They then had 67%, 60% and 51% nucleotide sequence similarity also to the bat-borne *mobatvirus,* Láibīn virus, identified in *Taphozous melanopogon* from China and Myanmar in 2015 and 2109. This data suggests the presence of a putative novel *Mobatvirus*, hereby designated Robina virus (ROBV), in Australian bats. In addition, a novel retrovirus was also identified and has been discussed elsewhere [48].

### Phylogeny

**Fig. 1:**
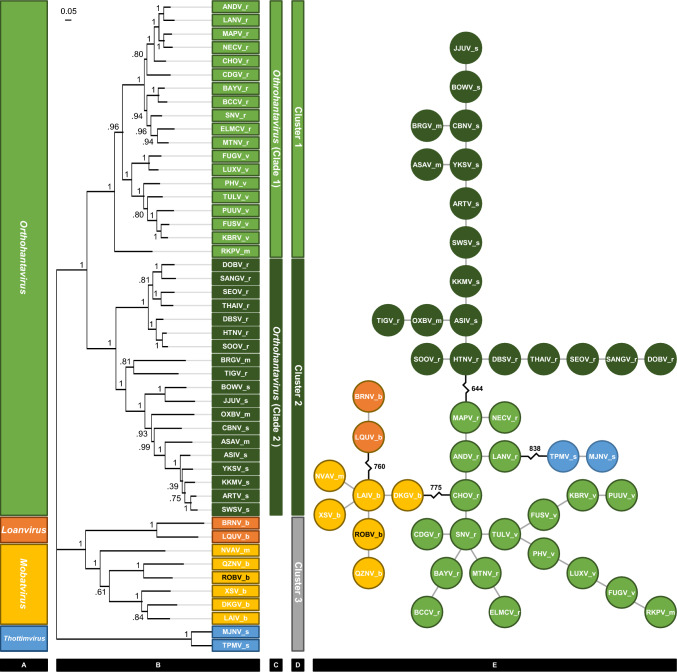
Analysis from the evolutionary relationship of a putative novel Australian hantavirus, Robina virus (ROBV) and 48 other classified hantaviruses performed using the concatenated amino acid sequence for the complete coding sequence of both the small and medium segments. **A** The current accepted genera. **B** and **C** A maximum-likelihood tree with clades representative of the current accepted genera and two smaller clades (clade 1 and clade 2), evident within *Orthohantavirus*. **D** Hierarchical clustering using a Bayesian methodology that produced three clusters (K = 3), the first, cluster 1 being identical to clade 1 from the maximum-likelihood analysis, the second, cluster 2 being identical to clade 2, and the third encompassing the remaining genera, *Loanvirus*, *Mobatvirus* and *Thottimvirus*. **E** A minimum spanning tree that connects hantaviruses by the minimum genetic distance (nucleotide differences), the greatest distance is between *Orthohantaviruses and Thottimvirus* (838 nucleotide differences), *Mobatviruses* and *Orthohantaviruses* (775 nucleotide differences), *Mobatviruses* and *Loanviruses* (760 nucleotide differences), and finally between the two *Orthohantaviruses* clades/clusters (644 nucletide differences)

Analysis of the evolutionary relationships of the concatenated amino acid sequences using maximum-likelihood analysis produced major clades that were identical to the existing genera, i.e. *Orthohantavirus*, *Loanvirus*, *Mobatvirus* and *Thottimvirus* (Fig. 1A and B). ROBV aligned most closely to QZNV within the *Mobatvirus* genus. In addition, it was evident that *Orthohantaviruses* could be subdivided into two clades (clade 1 and 2).

### Hierarchical clustering

Hierarchical clustering identified three discrete clusters (*K* = 3, Fig. 1D). Cluster 1 was identical to clade 1 (identified using maximum-likelihood analysis), cluster 2 was identical to clade 2 (also identified using maximum-likelihood analysis), and cluster 3 contained the three remaining genera *Loanvirus*, *Mobatvirus* and *Thottimvirus*. The posterior probability that each individual hantavirus belonged to its cluster was always one.

### Minimum spanning tree

Analysis indicated only one reliable tree (Fig. 1E). The genetic distances between different hantaviruses reflected grouping of the existing genera, i.e. the greatest distance was between *Thottimvirus* and *Orthohantavirus*, the second between *Mobatvirus* and *Orthohantavirus*, the third between *Mobatvirus* and *Loanvirus*. The smallest distance was between what was described as clade 1 and 2 when using maximum-likelihood analysis, or between cluster 1 and 2 when using hierarchical analysis, i.e. the subgroups in *Orthohantaviruses*.

### Experimental qRT-PCR

Although validation of the described qRT-PCR was not undertaken, an initial investigation suggested specificity of the assay with the only significant alignment returned from Primer-BLAST (NCBI) being the segment of interest (MK165655, E value 2^–9^), and no reaction with other viral RNA held at BSL. Between March 2018 and October 2023, a total of 495 bats were opportunistically screened for ROBV using the qRT-PCR. (qRT-PCR results, Supplementary material, and Table 1. Descriptive and univariate statistics). ROBV RNA was detected in the brain tissues of 21 bats. These included detections in 16 of 332 *P. alecto*, 3 of 75 *P. scapulatus* and 2 of 52 *P. poliocephalus*. There were no detections in 16 *P. conspicillatus* or 41 insectivorous bats.

### Confirmatory nested RT-PCR

Eight (8) samples (brain tissue) in which ROBV RNA was detected using an experimental qRT-PCR, with Ct values low enough to likely produce a PCR product (< 30), were sequenced using the confirmatory nested RT-PCR (data not shown). Seven of the sequences, six of which had 100% nucleotide sequence similarity and one with 99.2% similarity to the reference sequence (MK165655), were all derived from bats of the same species (*P. alecto*). The eighth sequence, which had the lowest nucleotide similarity (97.9%), was from a different bat species (*P. poliocephalus*). All nucleotide polymorphisms were synonymous. While the primer regions for the experimental qRT-PCR were conserved across all sequences, single nucleotide polymorphisms were identified in the secondary nested RT-PCR primers (T753C and A1119G) from the *P. poliocephalus* sequence.

### Statistics

Although no ROBV RNA was detected in the insectivorous bats, the total prevalence of ROBV RNA in *Pteropus spp.* was 4.2% (95% CI 2.8–6.4%, Table 1). Binomial modelling identified that there was substantial evidence to support the effect of year (*P* = 0.033), with a peak of detection in 2019 (5.6%, SE 1.8%) and 2020 (7.7, SE 2.9%). There was also moderate evidence to support the effect of season (*P* = 0.064), with peak detection in the cooler seasons, autumn (8.4%, SE 3.6) and winter (8.5%, SE 3.8%). There was minimal evidence to support the effect of region or species (*P* = 0.281 and 0.317).

### Virus isolation

No cytopathic effect was observed in either of the cell lines, and ROBV RNA was detected by qRT-PCR in successive passages at increasing Ct values, suggesting that no virus was isolated.

### Immunohistochemical staining

Immunohistochemical staining using polyclonal antibodies against both Puumala virus and Hantaan virus resulted in non-specific binding and did not resolve any features.

## Discussion

Robina virus (ROBV), species *Mobatvirus robinaense*, is a novel hantavirus first identified in the brain of a black flying fox (*Pteropus alecto*, family *Pteropodidae*), a large frugivorous bat. Whilst the complete coding sequence for this virus has been elucidated, and additional detections in other bats have been sequenced, the virus has not yet been isolated or visualised. Until this is achieved, it should be acknowledged that ROBV is a putative novel hantavirus.

The identification of Australia’s first reported putative hantavirus could extend the known southern range of hantaviruses into Australasia [5]. ROBV has the greatest amino acid sequence similarity (63%-80%) to another *Mobatvirus*, Quezon virus (QZNV), previously identified in the lung of a Philippine frugivorous bat (*Rousettus amplexicaudatus*, also family *Pteropodidae*) [13]. There is also a reported similarity of ROBV to Kiwira virus, recently identified in Angolan free-tailed bats (*Mops condylurus*, family *Molossidae*) from Tanzania and the Democratic Republic of Congo, however, this similarity is based on partial sequences of the S and L segments only and would benefit from additional sequence data, specifically from the S and M segments, to increase confidence in this relationship [12]. In addition to the identification of ROBV in *P. alecto*, at a prevalence of 5.3% (SE 1.6%, Table 1), ROBV RNA was also detected by experimental qRT-PCR in two other species of Australian bats from the same genus, *P. poliocephalus* (4.4%, SE 3.1%) and *P. scapulatus* (5.1%, SE 3.0%).

The equal prevalence of ROBV RNA in these three bat species could suggest either cross-species transmission or the presence of an ancient virus that evolved across all three species. Although small and acknowledging the limited sample size (n = 8), the equal prevalence of ROBV in our bats, and the differences in their nucleotide sequence similarity (100% and 99.2% between *P. alecto* vs. 97.9% when compared to *P. poliocephalus*) could suggest an evolutionary divergence of ROBV, with significant implications for both molecular biology and viral evolution. This divergence may also reflect species-specific adaptation, previously observed with *Orthohantaviruses* [14], and similar to what has been observed with Hendra virus, also in *Pteropus spp*. [49–51]. Efforts continue into acquiring a sequence from *P. scapulatus*, since this bat, which is a more distant relative of *P. alecto* and *P. poliocephalus* may host a more divergent hantavirus.

ROBV RNA was not detected in the fourth of Australia’s mainland *Pteropus spp.* (*P. conspicillatus*), or any insectivorous bats. However, due to the low sample size of *P. conspicillatus* (n = 16), and because of detections in the paraphyletic *P. alecto*, which has an overlapping home range, future detections of ROBV RNA in *P. conspicillatus* could be expected [52].

Discovery of hantaviruses are often made by PCR and the availability of complete coding sequence for the largest segment (L) is limited [5]. Exclusion of the L segment and concatenation of the S and M segments has been suggested as a suitable alternative for phylogenetic analysis until additional sequence information can be provided for classified viruses [25, 27]. Using only complete concatenated sequences from ROBV and 47 other previously classified hantaviruses (Table 1, Supplementary material), three types of phylogenetic analysis were performed, maximum likelihood, hierarchical, and minimum spanning tree (Fig. 1). All three analyses reliably predicted the current existing hantavirus genera, i.e. *Orthohantavirus*, *Loanvirus*, *Mobatvirus* and *Thottimvirus.* In addition, all three analyses predicted two subgroups (clades 1 and 2), within the genus *Orthohantavirus*. Whilst not proposing a new classification for the genus *Orthohantaviruses*, we do highlight these subgroups to aid further discussion into the source of ancient primordial hantaviruses and the forces driving their evolution [15, 26].

Previous studies have identified hantavirus in the brain, intestine, liver, lung, kidney and spleen from bats [6, 9–14, 24, 53]. This study opportunistically used brains predominantly, which had previously been tested at BSL for ABLV. However, in one instance (data not shown), when ROBV RNA was detected in the brain of a bat, it was also detected in the kidney and spleen of that bat, with a lower Ct in the spleen, a result similar to that previously reported by others [12]. Whilst acknowledging the limitations of using the brain as a screening tool, the convenience of using existing tissue, regularly submitted to BSL, allowed us to perform the largest temporal analysis of a putative hantaviruses in bats to date (6 years) and identified ecological factors potentially involved in infection dynamics.

The total prevalence of a putative ROBV in Australian bats, tested by an experimental qRT-PCR, was 4.2% (95%CI 2.8–6.4%, Table 1), similar to that of QZNV in *R. amplexicaudatus* (6.7%; 95% CI 1.2–29.8%) [13]. However, binomial modelling indicated that there was substantial evidence supporting an increase (*P* = 0.033) in the detection of ROBV RNA in bats in 2019 (5.6%, SE 1.8%) and 2020 (7.7%; SE 2.9%), declining to 1.1% (SE 1.1%) in 2022 and 2023. This infection dynamic is suggestive of a transient epidemic that occurs as ROBV, as with other bat borne viruses, are transmitted among bat populations [5, 13, 54, 55]. After recovery from infection, the subsequently developed population immunity will wane and allow reinfection with the virus producing the peaks observed in 2019 to 2020 [54]. Binomial modelling also identified moderate evidence (*P* = 0.064) that the prevalence of ROBV was greater in the cooler seasons, autumn (8.4%; SE 3.6%) and winter (8.5%; SE 3.8%), than spring (3.3%; SE 1.2%) and summer (1.4%; SE 1.0%). This seasonal variation in prevalence is a risk factor that has also been identified for another bat borne virus, specifically Hendra virus, also found in *P. alecto* [55, 56]. It is hypothesised that increased viral infection and excretion of Hendra virus is mediated by the physiological cost of thermoregulation, whilst others suggest that recent food shortages and displacement of bat populations into novel habitats predict the pronounced winter pulses [57, 58]. If season is significant, then these physiological and ecological factors may also underpin the infection dynamics that drive ROBV, and possibly other hantavirus infection in bats.

With the putative identification of Australia’s first hantavirus, future investigations are warranted. Firstly, previous surveillance in Australia detected antibodies to hantavirus in rodents, without ever identifying the responsible antigen [59]. It is now prudent to understand the role, if any, that ROBV may have played in generating those antibodies. Moreover, any future investigations should also include the surveillance of marsupials, given the detection of hantavirus in opossums in Brazil [10]. Secondly, ROBV was identified in the brain of a *P. alecto* during an investigation into the cause of neurological symptoms. The mild inflammatory changes observed in the brain's histology suggested a possible viral infection; however, it is premature to attribute encephalitis in this bat to ROBV. Determining any causal link between ROBV and the disease will require further investigations, which can only commence after the successful isolation of ROBV. These efforts continue at BSL.

## Conclusions

The discovery of ROBV, Australia's first reported putative hantavirus, marks a significant milestone in the understanding of hantaviruses, particularly in the context of Australian bat populations. The reporting of a complete coding sequence contributes to the advancement of hantavirus taxonomy and the development of diagnostic tools. Additionally, the identification of possible infection dynamics and seasonal variation raise intriguing questions about the physiological and ecological factors influencing hantaviruses in bats.

## Supplementary Information

Below is the link to the electronic supplementary material.

Supplementary file1 (XLSX 19 KB)

Supplementary file2 (DOCX 19 KB)

## Data Availability

Sequence data that support the findings of this study have been deposited in Genbank with the primary accession codes MK165653-MK165655. Epidemiological data has been submitted to Virus Genes in Supplementary data.

## Declarations

### Research involving human and animal participants

Ethical review and approval were waived for this study due to animals being submitted to the state for disease investigations unrelated to this study.
