# Supplementary material for: Identification and epidemiological analysis of a putative novel hantavirus in Australian flying foxes
Source: Virus Genes. 2024 Oct 11;61(1):71–80. doi: 10.1007/s11262-024-02113-3 (PMC11787259; doi:10.1007/s11262-024-02113-3)
Supplement: Supplementary file 2 — Supplementary file2 (DOCX 19 KB) [file 11262_2024_2113_MOESM2_ESM.docx]

Table 1. A list of classified hantaviruses (International Committee on Taxonomy of Viruses, <https://talk.ictvonline.org/taxonomy>), and their GenBank accession numbers, used to analyse the evolutionary relationship with a putative novel Australian hantavirus, Robina virus (ROBV).

| **Genus** | **Virus name** | **Abbreviation_host^1^** | **Small segment** | **Medium segment** |
| --- | --- | --- | --- | --- |
| *Loanvirus* | Brno virus | BRNV_b | KX845678 | KX845679 |
|  | Lóngquán virus | LQUV_b | JX465416 | JX465398 |
| *Mobatvirus* | Đakrông virus | DKGV_b | MG663534 | MG663535 |
|  | Láibīn virus | LAIV_b | NC_038514 | NC_038513 |
|  | Nova virus | NVAV_m | NC_034464 | NC_034470 |
|  | Quezon virus | QZNV_b | NC_034400 | NC_034393 |
|  | Xuân Sơn virus | XSV_b | KC_688335 | KU976427 |
|  | Robina virus | ROBV_b | MK165655 | MK165654 |
| *Orthohantavirus* | Andes virus | ANDV_r | NC_003466 | NC_003467 |
|  | Artybash virus | ARTV_s | MG888402 | MG913806 |
|  | Asama virus | ASAV_m | NC_038273 | NC_038274 |
|  | Asikkala virus | ASIV_s | NC_043070 | NC_043069 |
|  | Bayou virus | BAYV_r | NC_038298 | NC_038300 |
|  | Black Creek Canal virus | BCCV_r | NC_043075 | NC_043073 |
|  | Bowé virus | BOWV_s | NC_034405 | NC_034406 |
|  | Bruges virus | BRGV_m | NC_034394 | NC_034395 |
|  | Cano Delgadito virus | CDGV_r | NC_034528 | NC_034525 |
|  | Cao Bằng virus | CBNV_s | NC_034484 | NC_034474 |
|  | Choclo virus | CHOV_r | NC_038373 | NC_038374 |
|  | Dabieshan virus | DBSV_r | NC_038384 | NC_038383 |
|  | Dobrava-Belgrade virus | DOBV_r | NC_005233 | NC_005234 |
|  | El Moro Canyon virus | ELMCV_r | NC_038423 | NC_038424 |
|  | Fugong virus | FUGV_v | NC_034473 | NC_034466 |
|  | Fusong virus | FUSV_v | NC_038446 | NC_038447 |
|  | Hantaan virus | HTNV_r | NC_005218 | NC_005219 |
|  | Jeju orthohantavirus | JJUV_s | NC_034398 | NC_034404 |
|  | Kenkeme virus | KKMV_s | NC_034559 | NC_034565 |
|  | Khabarovsk virus | KBRV_v | NC_034527 | NC_034518 |
|  | Laguna Negra virus | LANV_r | NC_038505 | NC_038506 |
|  | Luxi virus | LUXV_v | NC_038530 | NC_038528 |
|  | Maporal virus | MAPV_r | NC_034566 | NC_034552 |
|  | Montano virus | MTNV_r | NC_034396 | NC_034397 |
|  | Necocli virus | NECV_r | NC_043409 | NC_043408 |
|  | Oxbow virus | OXBV_m | NC_043174 | NC_043176 |
|  | Prospect Hill virus | PHV_v | NC_038938 | NC_038940 |
|  | Puumala virus | PUUV_v | NC_005224 | NC_005223 |
|  | Rockport virus | RKPV_m | NC_038696 | NC_038694 |
|  | Sangassou virus | SANGV_r | NC_034526 | NC_034516 |
|  | Seewis virus | SWSV_s | EF636024 | KY651022 |
|  | Seoul virus | SEOV_r | NC_005236 | NC_005237 |
|  | Sin Nombre virus | SNV_r | NC_005216 | NC_005215 |
|  | Soochong virus | SOOV_r | AY675349 | AY675353 |
|  | Thailand virus | THAIV_r | NC_034555 | NC_034563 |
|  | Tigray virus | TIGV_r | KU934010 | KU934009 |
|  | Tula virus | TULV_v | NC_005227 | NC_005228 |
|  | Yákèshí virus | YKSV_s | NC_038704 | NC_038705 |
| *Thottimivirus* | Imjin virus | MJNV_s | NC_034558 | NC_034557 |
|  | Thottapalayam virus | TPMV_s | NC_010704 | NC_010708 |
